# Supplementary figures and images for: Intracellular DNA replication and differentiation of Trypanosoma cruzi is asynchronous within individual host cells in vivo at all stages of infection
Source: PLoS Negl Trop Dis. 2020 Mar 20;14(3):e0008007. doi: 10.1371/journal.pntd.0008007 (PMC7112235; doi:10.1371/journal.pntd.0008007)

## Slide 1
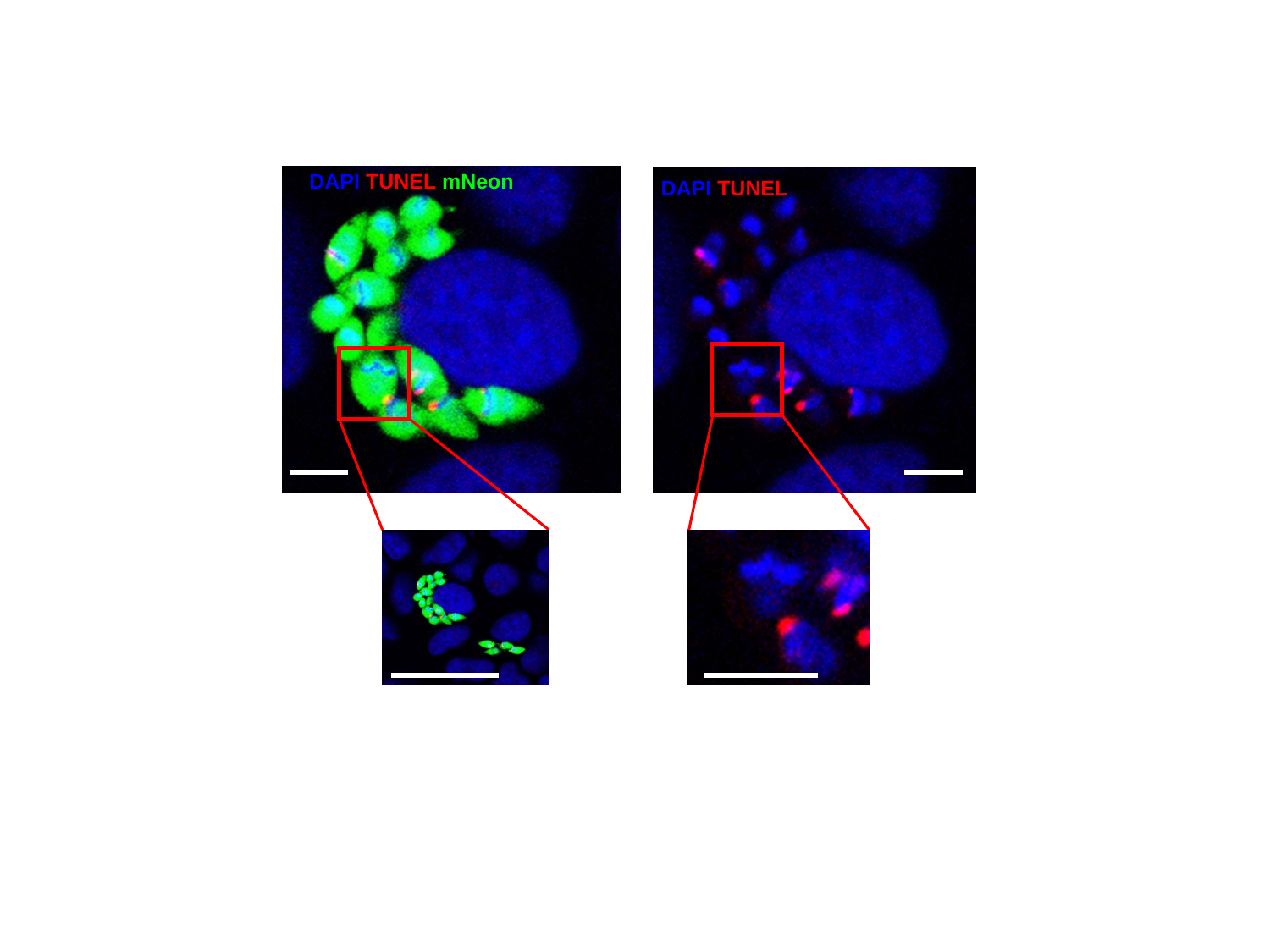

DAPI TUNEL mNeon
DAPI TUNEL

Supplement: S1 Fig — The parasite in the red box has completed kDNA replication and segregation, but not nuclear replication, and clearly shows that the segregated kinetoplasts no longer display TUNEL positivity. (PPTX) [file pntd.0008007.s001.pptx]

## Slide 1
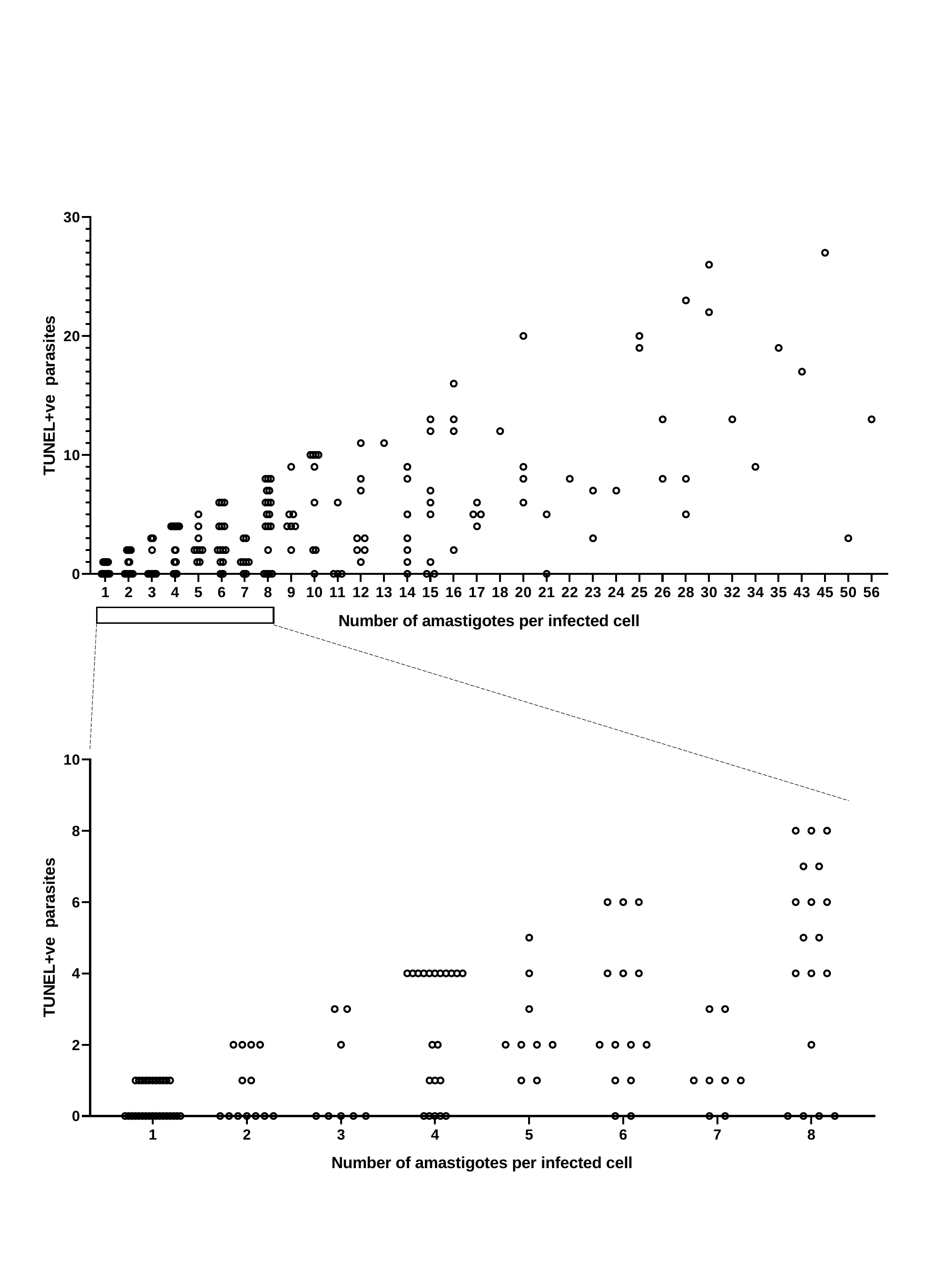

Supplement: S2 Fig — Each circle represents a single infected host cell. (a) All 200 infected cells from Fig 1D. (b) An expanded view of the area indicated by the box to allow clear visualisation of the host cell numbers. For cells infected with 1 amastigote, n = 28. (PPTX) [file pntd.0008007.s002.pptx]

## Slide 1
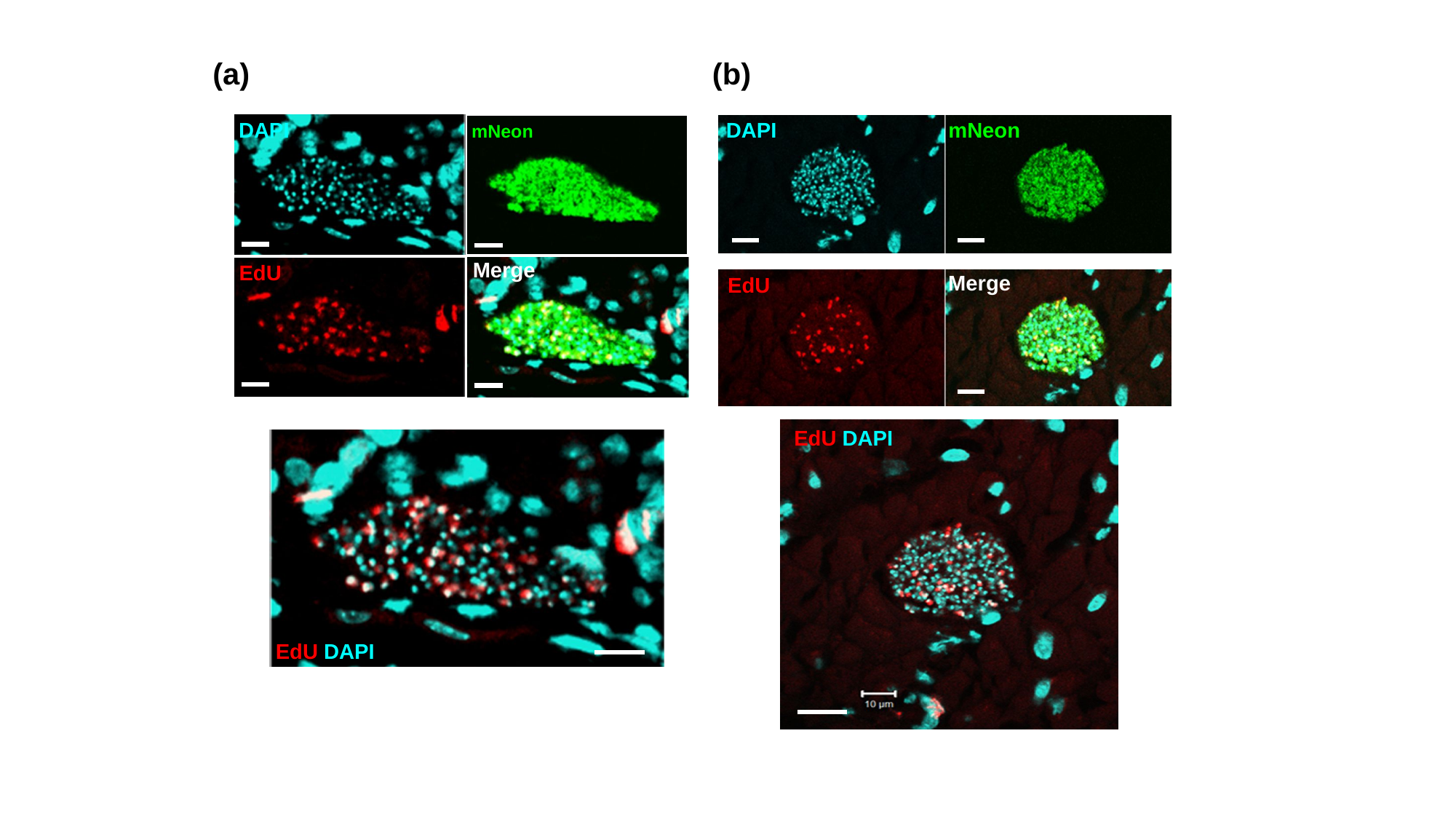

(a)
(b)
DAPI
DAPI
mNeon
mNeon
Merge
EdU
Merge
EdU
EdU DAPI
EdU DAPI

Supplement: S4 Fig — Replication of parasite DNA within mice infected by T. cruzi clone CL-Luc::Neon (Costa et al., 2018) was assessed after inoculating two EdU pulses 18 and 28 hours prior to tissue sampling (Experimental procedures). Parasites were located in histological sections by fluorescence (mNeon, green). a) DNA replication (red) in a chronic phase parasite nest (colon). The combined DAPI/EdU image illustrates the heterogeneity of parasite replication within the nest. Bar = 10 μm. b) Section from colon of mouse showing parasite nest. Upper panels show individual channels and a merged image. The lower panel shows DAPI and EdU channels only, allowing visualisation of the interspersed nature of EdU+ve amongst EdU-ve parasites. (a) and (b) are from different mice. Bars indicate 10 μm. (PPTX) [file pntd.0008007.s004.pptx]

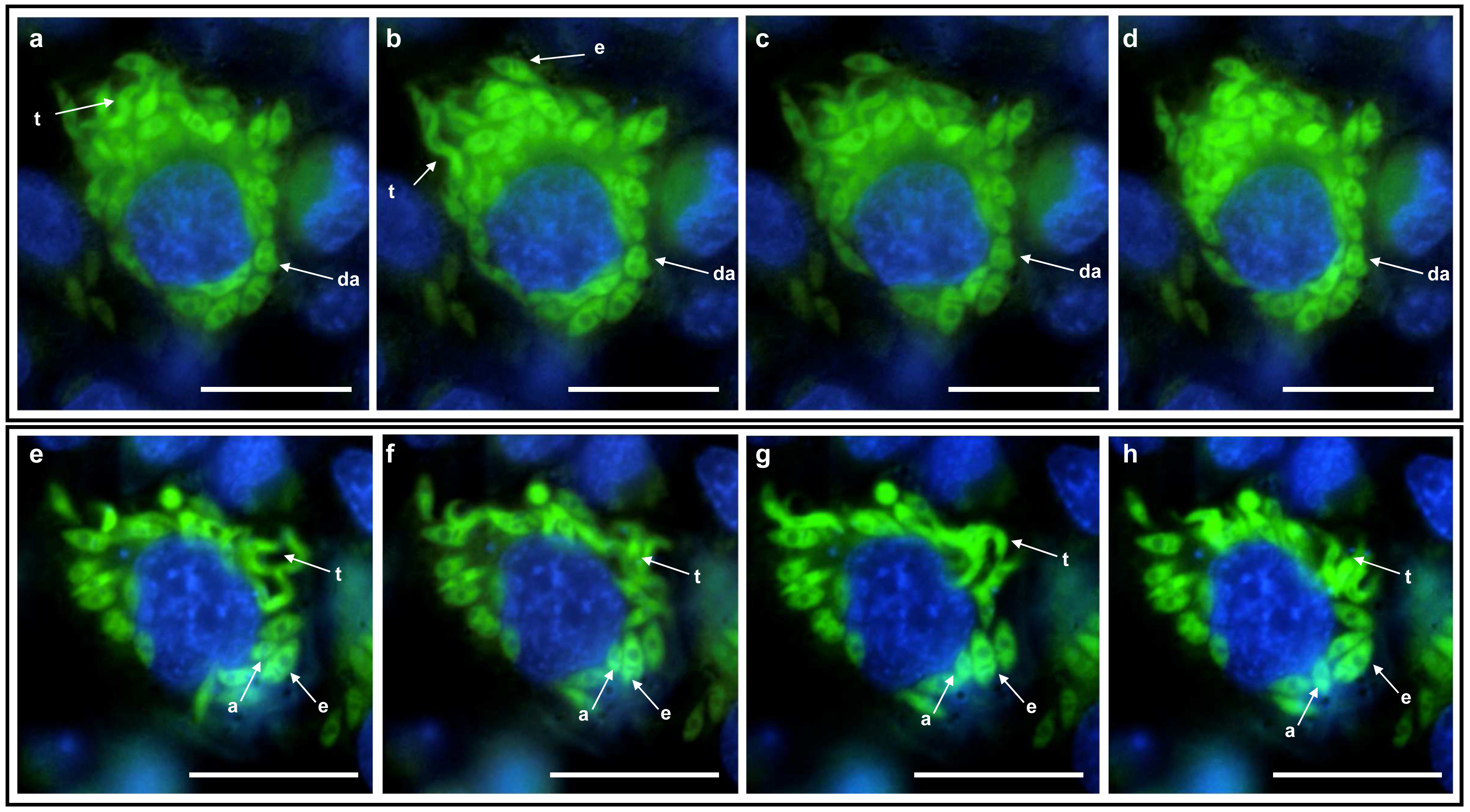

Supplement: S5 Fig — Each image shows an MA104 cell (blue, nucleus) 6 days after infection with T. cruzi (green) showing amastigotes (arrow a) dividing amastigotes (arrow da), epimastigote-like forms (arrow e) and trypomastigotes (arrow t) within the same cell. (a-d) sequential still images from S1 Movie, (e-h) sequential still images from S2 Movie. Bars indicate 20 μm. (TIF) [file pntd.0008007.s005.tif]
