# Supplementary material for: Intracellular DNA replication and differentiation of Trypanosoma cruzi is asynchronous within individual host cells in vivo at all stages of infection
Source: PLoS Negl Trop Dis. 2020 Mar 20;14(3):e0008007. doi: 10.1371/journal.pntd.0008007 (PMC7112235; doi:10.1371/journal.pntd.0008007)
Supplement: S3 Fig — (a) caecum, (b) rectum, (c) heart, (d) spleen and (e) lung. Images are from two individual mice. Bar = 10 μm. (PPTX) [file pntd.0008007.s003.pptx]

## Slide 1
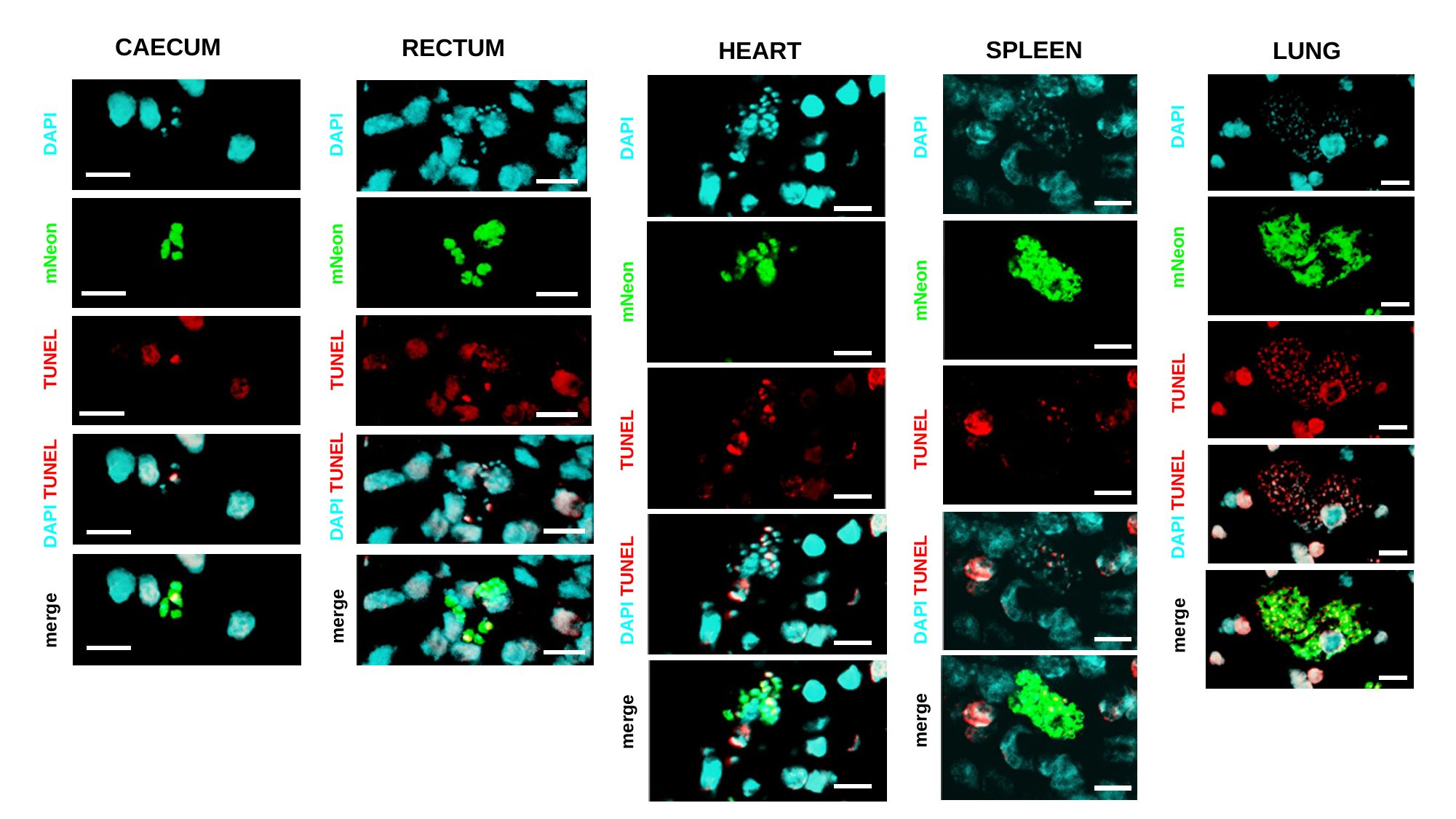

CAECUM
RECTUM
SPLEEN
LUNG
HEART
DAPI
DAPI
DAPI
DAPI
DAPI
mNeon
mNeon
mNeon
mNeon
mNeon
TUNEL
TUNEL
TUNEL
TUNEL
TUNEL
DAPI TUNEL
DAPI TUNEL
DAPI TUNEL
DAPI TUNEL
DAPI TUNEL
merge
merge
merge
merge
merge
